# Genome-Wide Thioredoxin System in *Cardamine hupingshanensis*: Role in Se Stress and Metabolism

**DOI:** 10.3390/biology14101404

**Published:** 2025-10-13

**Authors:** Yao Li, Huanqiu Xue, Yanke Lu, Zhixin Xiang, Zhi Hou, Yifeng Zhou, Qiaoyu Tang

**Affiliations:** 1 Hubei Key Laboratory of Biological Resources Protection and Utilization, Hubei Minzu University, Enshi 445000, China; 202330405@hbmzu.edu.cn (Y.L.);; 2 College of Biological and Food Engineering, Hubei Minzu University, Enshi 445000, China; 3 College of Forestry and Horticulture, Hubei Minzu University, Enshi 445000, China

**Keywords:** thioredoxin system, *Cardamine hupingshanensis*, redox regulation, Se metabolism, stress response

## Abstract

The thioredoxin system is crucial for maintaining redox balance and stress responses in plants, but its role in selenium hyperaccumulators remains poorly understood. To our knowledge, this study is the first to perform a genome-wide identification of the thioredoxin system in Se hyperaccumulator *Cardamine hupingshanensis*. *We* identified 74 *ChTRX* genes and 12 *ChTR* genes, among which *ChTRX* genes accounted for approximately 86.05% of the total identified thioredoxin system genes. Phylogenetic and structural analyses classified the ChTRXs into two types, typical (with the WCGPC active site) and atypical (with the XCXXC active site), with typical ChTRXs comprising about 48.65% and atypical ChTRXs about 51.35% of the total ChTRXs. Subcellular localization analysis revealed a diverse distribution, such as chloroplast, mitochondrion and cytoplasm. The chloroplast-localized ChTRXs are the most abundant, accounting for approximately 60% of all ChTRXs. Under Se stress, the expression of *ChTRX* genes exhibited significant tissue-specific differences: approximately 52.5% of *ChTRX* genes showed responsive expression in the roots, while only 31.25% responded in the leaves, suggesting that root-specific genes may play an important role in mitigating Se-induced oxidative damage. Through expression data and molecular docking analysis, we discovered that ChACHT4-1 can interact with the disulfide bonds of key Se metabolism related enzymes ChAPK and ChAPR, suggesting its potential reductive activity. Furthermore, we predicted stress-responsive ChTRXs regulated by multiple ChNTRs in TRX–TR regulatory pathway. Overall, our research indicates that the thioredoxin system influences Se metabolism in *C. hupingshanensis* through redox regulation, providing insights into the Se tolerance mechanisms of hyperaccumulating plants and offering perspectives for optimizing Se biofortification strategies in crops.

## Simple Summary

As a selenium hyperaccumulator plant, the role of the thioredoxin system in *Cardamine hupingshanensis* in selenium stress response and selenium metabolism remains unclear. Elucidating the function of this system is of important significance for understanding the mechanisms of plant selenium tolerance and optimizing selenium biofortification in crops. This study presents the first genome-wide identification of the thioredoxin system in *C. hupingshanensis*, aiming to investigate its functions under selenium stress and in selenium metabolism. In the present study, we identified 74 *thioredoxin* (*TRX*) genes and 12 *thioredoxin reductase* (*TR*) genes, which can be classified into different types and are widely distributed within cells. Under selenium stress, these genes exhibited tissue-specific expression patterns, and predictions suggest that some selenium stress-responsive genes regulate the redox processes of key enzymes involved in selenium metabolism. In summary, this study preliminarily demonstrates that the thioredoxin system in *C. hupingshanensis* may influence selenium metabolism through redox regulation, providing insights into the mechanisms of selenium tolerance in hyperaccumulator plants.

## 1. Introduction

**Figure 1:**
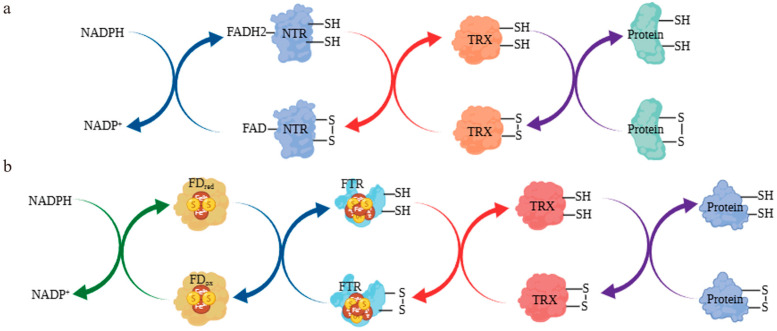
Redox Mechanism of the Plant Thioredoxin System. (**a**) Redox Mechanism Between TRX and NTR. (**b**) Redox Mechanism Between TRX and FTR.

The thioredoxin system is a crucial part of the enzymatic antioxidant defence, functioning through the coordinated action of thioredoxin (TRX) and thioredoxin reductase (TR) to support essential biological roles [1,2]. TRX, a small widespread protein, reduces -S-S- bonds in target proteins via its conserved Cys-X-X-Cys motif at the active site, converting them into -SH groups to influence protein structure, activity, or interactions [3]. Meanwhile, TR regenerates reduced TRX using NADPH or reduced ferredoxin (FD) [4]. TR generally exists as a homodimer containing an FAD cofactor and an NADPH-binding domain per subunit [5]. TR achieves high efficiency in mammalian mitochondria thanks to its C-terminal selenocysteine active site [6]. Although plant TR lacks Sec residues, it regulates redox through cysteine centres. The N-terminal domain binds FAD to facilitate electron transfer. At the same time, the C-terminal owns the NADPH-binding site and catalytic center, which may undergo allosteric regulation in plants to monitor redox states. This system functions as a molecular switch, enabling plants to adapt to environmental changes by regulating disulfide/thiol exchange in target proteins (Figure 1), thereby linking redox signal to metabolic processes.

As a core component of the antioxidant system, the thioredoxin system protects cells from oxidative damage by regulating the activity of antioxidant enzymes [7]. Specifically, TRX supplies electrons for the reduction of peroxides such as hydrogen peroxide (H_2_O_2_) and acts synergistically with glutathione to enhance glutathione peroxidase activity [8]. Meanwhile, TRX also reduces disulfide bonds in enzymes responsible for decomposing H_2_O_2_ and other peroxides, thereby reactivating them after catalytic cycles [9,10]. Under oxidative stress conditions in mammals, the heme cofactor in the active site of catalase undergoes overoxidation [11]. It becomes inactivated—a process achieved by converting hydrogen peroxide into water and oxygen, ultimately forming oxidised ferric heme [12]. Beyond antioxidant defense, TRX regulates key enzymes in various metabolic pathways via redox modifications: in photosynthesis, it activates Calvin cycle enzymes, such as Rubisco activase and fructose-1,6-bisphosphatase, through light-dependent reduction, ensuring synchronization between light reactions and carbon fixation in chloroplasts [13,14]; in lipid metabolism, it activates acetyl-CoA carboxylase to promote fatty acid synthesis [15], enhances fatty acid desaturase activity to maintain membrane fluidity [16], reduces lipoxygenase-mediated peroxidative damage [17], facilitates lipid mobilization via triacylglycerol lipase to provide energy [18], and coordinates phospholipase D-dependent lipid signaling; and in sulfur metabolism, it promotes the synthesis of cysteine and glutathione by reducing the oxidized cysteine residues in ATP sulfurylase, thereby enabling the enzyme to convert sulfate into organic sulfur [19]. This system may enhance sulfur uptake and allocation through two mechanisms: first, by regulating the activity of sulfur transporters at the post-translational level [20]; second, by potentially influencing the DNA-binding capacity of the transcription factor SLIM1 through mediation of the intracellular redox state [21]. Additionally, TRX influences the plant’s antioxidant capacity at the transcriptional level by regulating transcription factors such as HSFs, STOP1, NPR1, and NAC family members [22,23,24,25].

Selenium (Se) is not only an essential trace element for humans and other animals, but also an important element for plant growth and development, with its effects following a dose-effect relationship. In humans, Se deficiency increases the risk of Keshan disease and certain metabolic disorders, while excessive Se intake can lead to toxic reactions [26,27]. In plants, supplementation with low concentrations of Se can promote growth, enhance antioxidant defense, and improve stress resistance, whereas high concentrations of Se may induce oxidative stress, inhibit photosynthesis, and disrupt metabolic processes [28,29]. Plant-derived Se is an essential source of Se nutrition for humans, as plants can convert inorganic Se in soil into bioavailable organic Se, helping humans maintain an appropriate Se level and thereby reducing the risks associated with Se deficiency. Se hyperaccumulator plants (*Cardamine hupingshanensis* (*C. hupingshanensis*) [30], *Astragalus bisulcatus* [31], and *Stanleya pinnata* [32]) have evolved unique Se tolerance and accumulation mechanisms, with Se concentrations in their bodies reaching 100–1000 times those in non-accumulator plants. These plants convert toxic inorganic Se into organic Se forms with lower toxicity and sequester them in vacuoles to minimise cellular damage [33]. Due to the similarity in chemical structure between Se and sulfur, their metabolic pathways overlap; thus, the thioredoxin system, a key regulator of sulfur metabolism, may also regulate Se assimilation and Se tolerance in hyperaccumulator plants. Se pollution caused by mining, agricultural runoff, and industrial activities threatens ecosystems and human health [34]. Therefore, research on Se hyperaccumulator plants such as *C. hupingshanensis* has dual value: on one hand, such research can reveal the molecular mechanisms underlying the extreme Se tolerance of plants; on the other hand, it can not only provide insights for formulating Se biofortification strategies but also offer references for advancing phytoremediation technologies.

*C. hupingshanensis*, discovered in the Wuling Mountain region, is a hyperaccumulator plant of significant research value, likely possessing unique mechanisms for selenium absorption, transport, metabolism, and tolerance [35,36,37]. While genes involved in selenium metabolism in *C. hupingshanensis* have been reported in previous studies, its thioredoxin system remains uninvestigated, and its regulatory role in selenium metabolic pathways is still unknown. To better understand the selenium tolerance mechanisms in *C. hupingshanensis*, this study focuses on characterizing the molecular features and functional roles of the thioredoxin system in this species. Through bioinformatics analysis, we identified members of the *ChTRX* and *ChTR* families. Sequence alignment and conserved domain prediction revealed their structural characteristics, while phylogenetic analysis explored evolutionary relationships and potential functional divergence. Protein structure modelling predicted the 3D conformations of ChTRX and ChTR proteins. Using quantitative real-time PCR (qRT-PCR), we analyzed the expression patterns of *ChTRXs* and *ChTRs* under Se treatment with different concentrations and exposure durations and screened out members responsive to Se stress. Additionally, we investigated the interactions between the thioredoxin system of *C. hupingshanensis* and Se metabolized enzymes, APK and APR, and focused on binding energies, sites, and key residues to elucidate redox regulation mechanisms. Collectively, this research aims to explain the role of the thioredoxin system in regulating Se metabolism in plants, laying the groundwork for enhancing Se nutrition and stress tolerance in crops.

## 2. Materials and Methods

### 2.1. Identification of ChTRX and ChTR Genes in C. hupingshanensis

The genome and annotation files of *C. hupingshanensis* were obtained from the Genome Warehouse BIG Data Centre (https://ngdc.cncb.ac.cn/gwh/, accessed on 14 August 2024) with the accession number PRJCA005533. To identify TRX and TR gene members in *C. hupingshanensis*, nucleotide and protein sequences of *AtTRX* and *AtTR* genes were downloaded from The Arabidopsis Information Resource (https://www.arabidopsis.org/, accessed on 14 August 2024) as reference sequences. The most similar protein sequences of *ChTRXs* and *ChTRs* were extracted using the BlastZone function in TBtools-II software [38]. To further validate the accuracy of extracted ChTRX and ChTR protein sequences from the NCBI BLAST+2.16.0 website (https://blast.ncbi.nlm.nih.gov/Blast.cgi, accessed on 15 August 2024), conserved domains were analyzed using CD-Search (https://www.ncbi.nlm.nih.gov/Structure/bwrpsb/bwrpsb.cgi, accessed on 17 August 2024).

### 2.2. Physicochemical Properties and Phylogenetic Analysis of ChTRXs and ChTRs

The molecular weight (MW), isoelectric point (pI), and other physicochemical properties of ChTRX and ChTR proteins were predicted using ExPASy (https://web.expasy.org/protparam/, accessed on 20 August 2024). Subcellular localization was predicted using WoLF PSORT (https://wolfpsort.hgc.jp/, accessed on 20 August 2024). To investigate the evolutionary relationships of the TRX and TR families, protein sequences from various plants were downloaded from NCBI (https://www.ncbi.nlm.nih.gov/, accessed on 20 August 2024), including 41 TRX sequences from *Arabidopsis thaliana* (*A. thaliana*), 151 from soybean, and 100 from wheat, as well as 6 TR sequences from *A. thaliana*, 16 from soybean, 10 from wheat, 6 from peanut, and 11 from rice. The *ChTRX* and *ChTR* gene sequences were aligned with those from other plants using MEGA11 software, and phylogenetic trees were constructed using the maximum likelihood (ML) method, with the analysis performed in MEGA 11. The optimal amino acid substitution model was selected based on the Bayesian Information Criterion (BIC), ultimately identifying the Jones-Taylor-Thornton (JTT) model as the most suitable. During the analysis, sequence gaps were handled using the ‘use all sites’ strategy, meaning that all sites containing gaps and missing data were included. Branch support was assessed with 1000 bootstrap replicates, and all other parameters were set to their default values. The resulting multi-species phylogenetic trees were visualized and annotated using the iTOL website (https://itol.embl.de/, accessed on 30 August 2024).

### 2.3. Chromosomal Localization and Domain Analysis of ChTRXs and ChTRs

The chromosomal positions of *ChTRX* and *ChTR* genes were determined by analyzing the genome annotation file of *C. hupingshanensis*. These positions were visualized using the advanced gene location visualization function in TBtools and chromosome density data. Based on the genome annotation file and conserved domain information obtained from NCBI CD-Search (https://www.ncbi.nlm.nih.gov/Structure/bwrpsb/bwrpsb.cgi, accessed on 5 September 2024), the gene structures of *ChTRX* and *ChTR* were illustrated using the Gene Structure View (Advanced) function in TBtools. Conserved motif analysis of TRX/TR family protein sequences was performed using the Simple MEME Wrapper function in TBtools-II software. The parameters were set as follows: the number of motifs was 15, the motif width range was restricted to 6–50 amino acids, the maximum number of iterations was 5000, and the background model used was the ‘zero-order’ model. The motif filtering criterion was an E-value ≤ 0.05, and only conserved motifs meeting this threshold were retained for subsequent analyses to ensure the reliability of the identified results. The intron-exon structures of *ChTRX* and *ChTR* were extracted from the GFF file and visualised using TBtools. ChTRX/TR protein sequences were aligned using the ClustalW algorithm in MEGA 11. All alignment results were visualized with ESPript 3.0 (https://espript.ibcp.fr/ESPript/cgi-bin/ESPript.cgi, accessed on 9 September 2024) under default parameters.

### 2.4. Plant Materials and Sample Preparation

*C. hupingshanensis*, a Se hyperaccumulator endemic to the Wuling Mountain region of Hubei Province, China, is an essential model for studying Se hyperaccumulation mechanisms. Seeds obtained from the Key Laboratory of Hubei Minzu University in Enshi City were germinated and grown in a controlled climate chamber maintained at 22 ± 1 °C with a 16 h photoperiod and light intensity of 1500 mol^−2^ ms^−1^. Forty-eight uniformly grown seedlings were selected, carefully rinsed with clean water to remove sand from the root systems, and transferred to disposable plastic cups containing Hoagland’s nutrient solution for a two-day acclimatisation period. The Hoagland’s nutrient solution primarily consists of ammonium dihydrogen phosphate, potassium nitrate, calcium nitrate, magnesium sulfate, phosphates, and other components, with a pH range of 5.0–6.5. After acclimatisation, the seedlings were exposed to Se solutions at different concentrations for 24 h, with three replicates designed for each treatment group. The experimental design included a blank control (0 μg Se·L^−1^) to account for background Se interference, a low-concentration treatment (100 μg Se·L^−1^) simulating near-natural seleniferous conditions slightly above typical aquatic Se levels in high-Se regions to investigate basic adaptation mechanisms and gene expression changes, and a high-concentration treatment (80,000 μg Se·L^−1^) creating extreme Se stress far exceeding normal growth conditions to analyze tolerance thresholds, emergency response strategies, and physiological metabolic adaptations, thereby elucidating the specialized mechanisms of Se hyperaccumulation [39]. We were treated with sodium selenite (Na_2_SeO_3_) at concentrations of 100 and 80,000 μg Se·L^−1^. Leaves and roots samples were collected at 0, 3, 6, 9, 12, and 24 h after treatment, flash-frozen in liquid nitrogen, and stored at −80 °C. RNA extraction was completed within 72 h.

### 2.5. Gene Expression Analysis

Total RNA was extracted from root and leaf samples using the TransZol™ Up Plus RNA Kit (TransGen Biotech, Beijing, China). RNA concentration was measured using NanoDrop 2000 (Thermo Fisher Scientific, Waltham, MA, USA), and RNA integrity and genomic DNA contamination were assessed by 1.0% agarose gel electrophoresis. Following the manufacturer’s instructions, the first-strand cDNA was synthesized from 2 μg RNA using the HiScript III RT SuperMix (gDNA wiper) Kit (Vazyme, Nanjing, China). Real-time qPCR was performed on ABI StepOne Plus system (Thermo Fisher Scientific, Waltham, MA, USA) using the Hieff qPCR SYBR Green Master Mix (Low Rox Plus) Kit (Yeasen Biotechnology, Shanghai, China). Each reaction mixture (10 μL) contained 5 μL of Hieff qPCR SYBR Green Master Mix, 1 μL of cDNA, 0.2 μL of forward primer (10 μmol L^−1^), 0.2 μL of reverse primer (10 μmol L^−1^), and 3.6 μL of RNase-free ddH_2_O. The qPCR amplification protocol was as follows: initial denaturation at 95 °C for 5 min, 40 cycles of denaturation at 95 °C for 10 s, annealing at 57–59 °C (gene-specific), and extension at 72 °C for 20 s. All genes’ cycle threshold (Cq) values ranged from 18.65 to 29.63. The ChActin gene was used as an internal reference for normalization, and relative gene expression was calculated using the 2^−ΔΔCT^ method [40]. Each reaction contained cDNA equivalent to 100 ng of total RNA. Melting curve analysis was performed under the following conditions: 95 °C for 15 s, 60 °C for 1 min, followed by a gradual temperature increase from 60 °C to 95 °C at a rate of 0.5 °C/s, with continuous fluorescence monitoring to verify amplification specificity. All reactions were performed in triplicate. After averaging the triplicate datasets, the 0 h time point was used as the control for normalization. The normalized data were visualized using the Polar Heatmap Dendrogram tool available in the APPs package of OriginPro 2025b, with all parameters set to their default options. Primers used for qRT-PCR analysis of *ChTRXs* and *ChTRs* are listed in Table S5.

### 2.6. Protein Modelling and Validation of ChTRX and ChTR

The amino acid sequences of ChTRX and ChTR proteins were submitted to SOPMA (Self-Optimised Prediction Method with Alignment; https://npsa-prabi.ibcp.fr/cgi-bin/npsa_automat.pl?page=/NPSA/npsa_sopma.html, accessed on 2 March 2025) for secondary structure prediction. AlphaFold 3 (https://alphafoldserver.com/, accessed on 10 March 2025) was used to predict the three-dimensional (3D) structural model based on the amino acid sequence, employing the monomer prediction pipeline during the prediction process. Five iterations of model generation were performed, and the model with the highest confidence was selected for subsequent analysis. Key metrics were applied to validate the quality of the final 3D model: the predicted template modelling score (pTM) was used to evaluate the overall reliability of the single-chain structure, where a pTM value > 0.5 indicates that the predicted structure is highly likely to resemble the actual structure [41,42]. During prediction, five iterations of model generation were performed, and the model with the highest confidence was selected. Additionally, the interface predicted template modelling score (ipTM) was utilized as a supplementary validation parameter.

### 2.7. Ligand Preparation and Protein Docking

The ligand protein structures ChAPK and ChAPR were obtained using the same method as the donor protein ChTRXs. The predicted ligand and donor protein structures were opened in PyMOL, where small molecules and water molecules were removed. For protein-protein docking, we first preprocessed the proteins using Maestro, modifying the two cysteine residues in the reduced-state protein to their oxidized state. Then, we uploaded the two proteins to the HADDOCK2.4 web server (https://rascar.science.uu.nl/haddock2.4/, accessed on 15 March 2025) and selected the docking sites based on literature evidence (the active site between TRX and TR is the CXXC motif, and the docking sites for APK/APR are the two critical cysteine residues), with other parameters set to default. The optimal conformation was screened using the z-value (a smaller z-value indicates a more reliable complex), combined with known functional site screening. After docking completion, the top 10 binding models were downloaded and reopened in PyMOL to analyse the docking sites visually. The structure of the ligand molecule FADH2 was retrieved from the PubChem database (https://pubchem.ncbi.nlm.nih.gov/, accessed on 5 April 2025), and its three-dimensional structure was converted to PDB format using Open Babel 3.1.1 software [43]. A protein model was constructed based on the amino acid sequence of ChTR, and the active sites of the protein were predicted using the Prankweb platform (https://prankweb.cz/, accessed on 2 April 2025). Molecular docking predictions were performed between the oxidised ChTR protein and the ligand compound using AutoDock 4.2.6 and AutoDock Vina v1.2.x software, with Gasteiger charges applied to calculate protein–ligand interaction energies [44]. Reducing equivalents are transferred from NADPH to FAD via the electron transport chain, reducing FAD to FADH2. These reducing equivalents are further transferred to the redox centre of NTR, which contains active-site disulfide bonds, inducing conformational changes and conferring reducing capability. The feasibility of this reaction was evaluated based on the Gasteiger charge–based analysis.

### 2.8. Statistical Analysis

Statistical analysis was conducted using GraphPad Prism software (version 9.3.0). One-way analysis of variance (one-way ANOVA) was applied to compare gene expression levels at different time points under the same selenium treatment concentration. All experiments were performed with three independent replicates, and the results are presented as mean ± standard deviation (SD). Post hoc comparisons between groups were carried out using Fisher’s Least Significant Difference (LSD) test. Differences were considered statistically not significant at *p* > 0.05 (ns), significant at 0.01 < *p* ≤ 0.05 (*) and highly statistically significant at *p* ≤ 0.01 (**).

## 3. Results

### 3.1. Identification and Analysis of ChTRX and ChTR Gene Families

**Figure 2:**
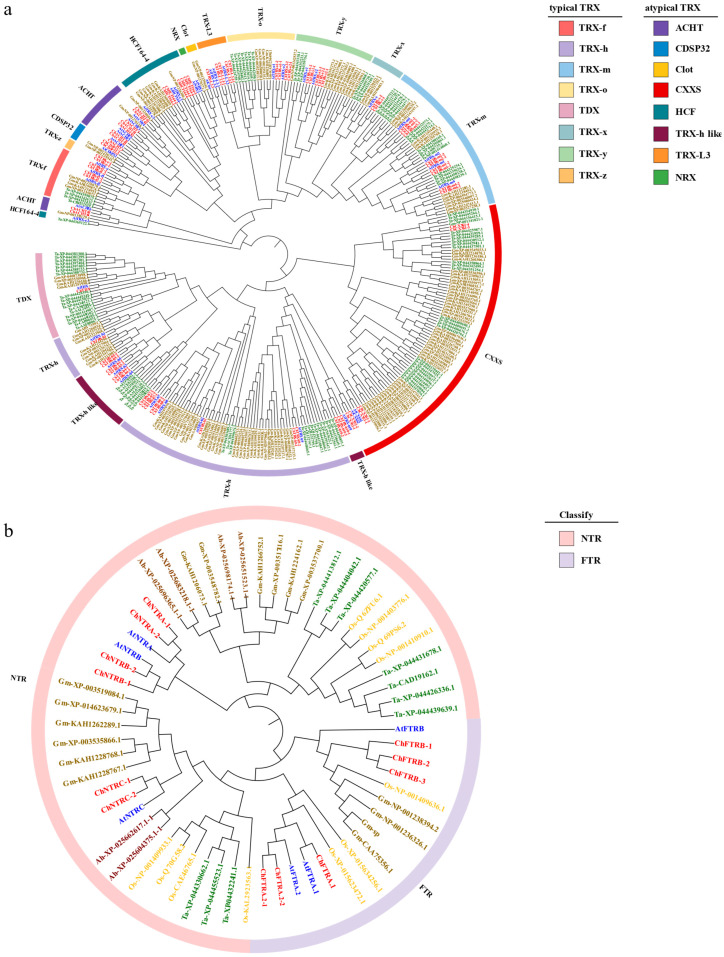
Phylogenetic analysis of the *ChTRX* and *ChTR* gene families. (**a**) Multi-species phylogenetic analysis of *TRXs*. (**b**) Multi-species phylogenetic analysis of *TRs*. At: *Arabidopsis thaliana*; Ch: *Cardamine hupingshanensis*; Ta: *Triticum aestivum*; Gm: *Glycine max*; Os: *Oryza sativa*; Ah: *Arachis hypogaea* L.

To investigate the redox regulatory mechanism of the thioredoxin system in *C. hupingshanensis*, we identified *TRX* and *TR* gene families from its genome (National Genomics Data Centre, accession PRJCA005533). We analyzed the physicochemical properties of their encoded proteins. Comparative genomic analysis with *A. thaliana* revealed 74 *ChTRX* genes, including 36 typical isoforms with the conserved WCGPC active site and 38 atypical isoforms containing variant XCXXC motifs, alongside 12 *ChTR* genes (Tables S1 and S2). Following the classification of *AtTRX* and *AtTR* [45], *ChTRX* and *ChTR* genes were categorized accordingly, and phylogenetic analysis indicated distinct evolutionary groupings (Figure 2). Comprehensive details about gene and protein characteristics, including amino acid count, theoretical pI, molecular weight, and additional properties, are listed in Tables S1–S3.

TRXs are small proteins widespread in prokaryotes and eukaryotes. Compared to *A. thaliana*, ChTRX proteins generally contain more amino acids, with molecular weights ranging from 7.86 kDa for ChTRX-h1 to 21.60 kDa for ChTRX-o1-1. Among the 74 ChTRXs, 66.2% (corresponding to 49 proteins) are weakly alkaline; 52.7% are predicted to be stable in vitro, and 83.8% are classified as hydrophilic. Subcellular localization predictions suggest that most ChTRXs reside in chloroplasts, while 18.9% localize in mitochondria, 16.2% in cytoplasm, 8.1% in endoplasmic reticulum, and 6.8% in the nucleus. In contrast, ChTRs exhibit amino acid counts and sizes comparable to those in *A. thaliana*. Excluding ChFTRA.1, the remaining 11 ChTRs range from 112 to 662 amino acids, with molecular weights varying between 12.59 kDa for ChFTRB-3 and 73.21 kDa for ChFTRA.2-1. Approximately 66.7% of ChTRs are alkalinous and 33.3% are acidic. Regarding stability, 58.3% are predicted to be unstable in vitro, while 100% are hydrophilic.

### 3.2. Phylogenetic Analysis of ChTRX and ChTR Gene Families

To elucidate the evolutionary relationships and functional predictions of *ChTRXs* and *ChTRs* in *C. hupingshanensis*, we constructed phylogenetic trees using homologous genes from multiple plant species. For *TRX* genes, four species were included: *C. hupingshanensis*, *A. thaliana*, *Glycine max*, and *Triticum aestivum*. For *TR* genes, six species were analyzed: *C. hupingshanensis*, *A. thaliana*, *G. max*, *T. aestivum*, *Oryza sativa*, and *Arachis hypogaea*. The phylogenetic analysis classified the 74 *ChTRX* genes into two major subclasses: 36 typical *TRXs* and 38 atypical *TRXs* (Figure 2a). The typical *TRXs* were further divided into eight types, including *TRX-f*, *TRX-x*, *TRX-y*, *TRX-z*, *TRX-m*, *TRX-o*, *TRX-h*, and *TDX*. The atypical *TRXs* also comprised eight types: *TRX-h-like*, *CDSP32*, *ACHT*, *TRX-like3*, *HCF164*, *CXXS*, *Clot*, and *NRX*. Comparative phylogeny revealed that *ChTRX* homologs do not exhibit one-to-one orthology with those in *A. thaliana*, but instead show one-to-many relationships, implying that gene diversification correlates with evolutionary divergence. Notably, within the *CXXS* subfamily, *ChCXXS1-3* and *ChCXXS1-4* are evolutionarily distant from other members, suggesting an ancient origin. Similarly, *ChHCF164-3* to *ChHCF164-6* form a clade distinct from *ChHCF164-1* and *ChHCF164-2*.

For the *TR* gene family, a multi-species phylogenetic tree incorporating both monocot and dicot species resolved the 12 *ChTR* genes into two subgroups, *NTR* and *FTR*, each containing six genes (Figure 2b). Interestingly, the plastid-localized *NTRC* did not cluster with *NTRA* or *NTRB*, but grouped closely with *FTR*, indicating more substantial sequence similarity and a closer evolutionary relationship between *NTRC* and *FTR*. Cross species comparison demonstrated that *ChTRs* share the highest phylogenetic affinity with *AtTRs*, consistent with their close taxonomic relationship within the Brassicaceae family and shared dicot lineage.

### 3.3. Chromosomal Localization and Domain Analysis of ChTRX and ChTR Gene Families

**Figure 3:**
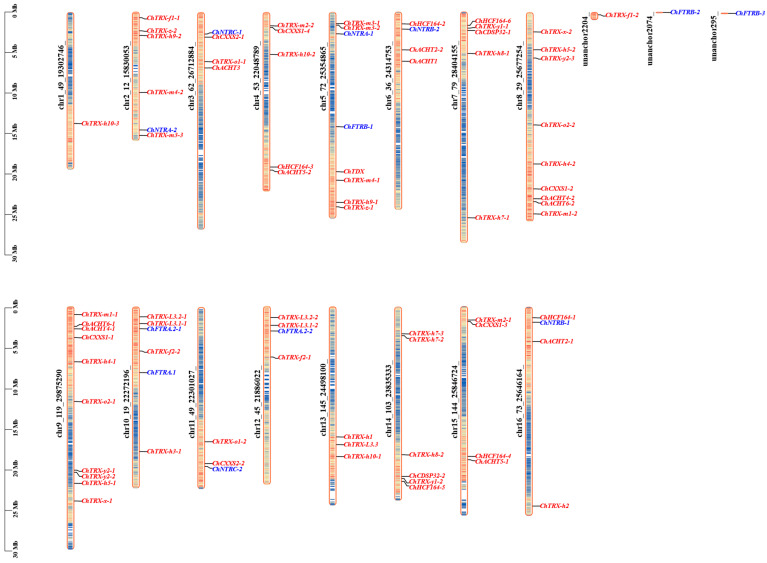
Chromosomal localization of the *ChTRX* and *ChTR* gene families. *ChTRXs* are marked in red, and *ChTRs* are marked in blue.

The *ChTRX* and *ChTR* genes are distributed across all 16 chromosomes of *C. hupingshanensis*, with no evidence of tandem gene duplication, as shown in Figure 3. The distribution of *ChTRX* genes is highly uneven: chromosome 9 contains the highest number, with 10 genes, followed by chromosome 8 with 9 genes. Among the remaining chromosomes, approximately 12.5% carry 4 to 6 *ChTRX* genes, while 25% contain 3 genes. Notably, chromosome 1 carries only one *ChTRX* gene. Two genes, *ChTRX-o1-2* and *ChCXXS2-2*, are uniquely located on chromosome 2. The 12 *ChTR* genes are also unevenly distributed, with most chromosomes harboring one or two genes. Genome annotation also identified several *ChTRX* and *ChTR* genes on unnamed chromosomal segments, which may correspond to incompletely assembled regions.

**Figure 4:**
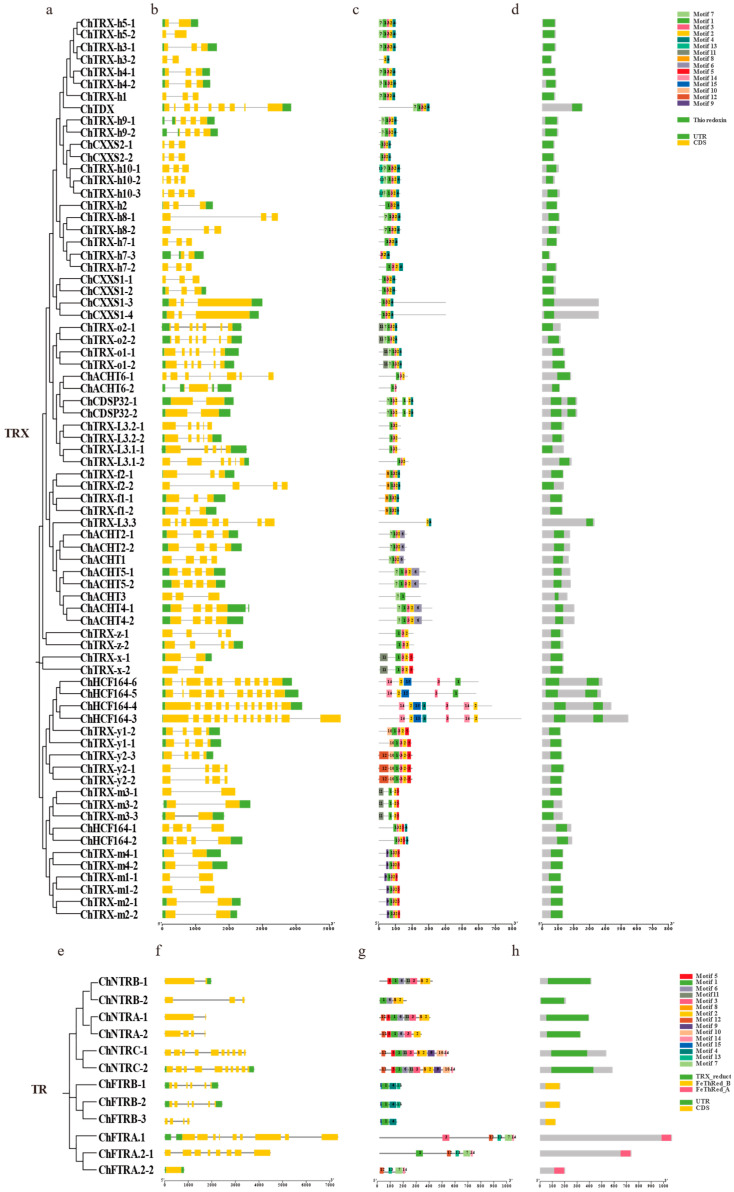
Phylogenetic tree, gene structure, conserved motifs, and structural domains of *ChTRX* and *ChTR* gene families. (**a**,**e**) Phylogenetic tree of *ChTRXs* and *ChTRs*. (**b**,**f**) Intron and exon structures of *ChTRX* and *ChTR* genes, exons and introns are represented by green boxes and black lines, respectively. (**c**,**d**,**g**,**h**) Conserved motifs and domains of ChTRX and ChTR; Different conserved motifs and domains are marked with distinct colours.

To examine structural conservation and divergence, we constructed maximum likelihood phylogenetic trees. We analyzed conserved motifs, domains, and gene structures of ChTRX and ChTR proteins (Figure 4). Gene structure visualization using TBtools revealed that homologous genes within the same phylogenetic group share highly similar intron–exon architectures and UTR distributions, suggesting strong functional conservation within subclades and noticeable divergence between them (Figure 4b,f). MEME suite analysis identified 15 conserved motifs across the proteins (Figure 4c,g). Motif 1, corresponding to the TRX active site, is present in 89.2% of ChTRX proteins. Several subfamily-specific motifs were detected: Motif 8 is unique to ChTRX-f; Motif 10 to ChTRX-y; Motif 11 to ChTRX-x, ChTRX-o, and ChTRX-m3; and Motif 9 is specific to other members of the ChTRX-m clade, indicating functional diversification. Among ChTR proteins, Motif 1 is the most widespread, and Motif 11 localizes to the active site of ChNTRs, while Motifs 7 and 13 are associated with ChFTR active sites. Domain analysis via NCBI CD Search confirmed that all ChTRX proteins contain the thioredoxin domain. In contrast, ChTR proteins carry the thioredoxin reductase domain, with specific isoforms also containing the FTR domain. The *FTR* gene encodes distinct α-chain (FTRA) and β-chain (FTRB) subunits, which utilize FD as an electron donor (Figure 4d,h).

### 3.4. Synteny and Evolutionary Analysis of ChTRX and ChTR Gene Families

**Figure 5:**
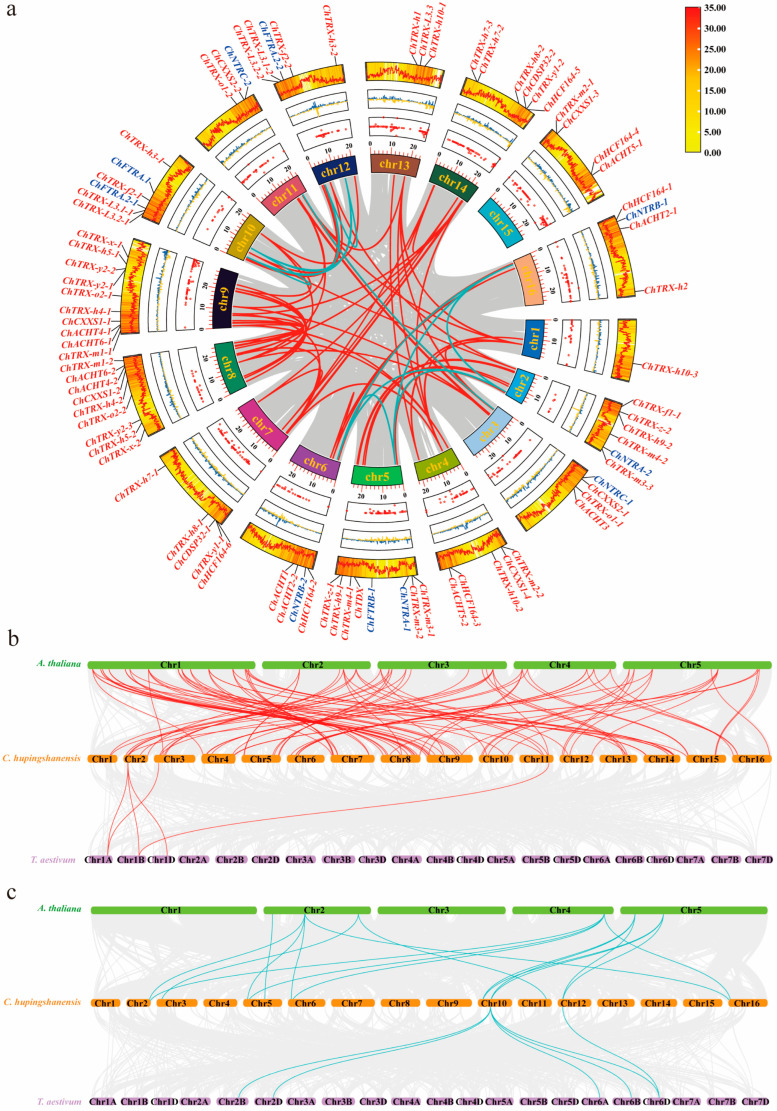
Synteny and Evolutionary Analysis of *ChTRX* and *ChTR* Gene Families. (**a**) Intra-genomic collinearity map of *ChTRXs* and *ChTRs*. In the Circos plot, progressing from inner to outer rings, the grey lines in the background represent collinear regions within the *C. hupingshanensis* genome. In contrast, the red lines highlight collinear gene pairs specifically within the *ChTRXs* and *ChTRs*. The figure also includes a dot plot showing N ratio distribution, a line plot of GC skew, a heatmap of gene density distribution, and a line plot depicting GC content variation. The labels indicate gene families, with *ChTRXs* marked in red and *ChTRs* in blue. (**b**,**c**) Synteny analysis of *TRX* and *TR* genes among *C. hupingshanensis*, *A. thaliana*, and *T. aestivum*. Red and cyan lines represent collinear pairs of *TRX* and *TR* genes, respectively, while grey lines denote all syntenic blocks within the genomes.

Gene duplication is a crucial process that fosters genomic innovation and the development of new traits and capabilities in organisms [46]. The redundancy created by duplication enables gene copies to acquire new functions through mutation and natural selection, enhancing the genome’s coding capacity and regulatory complexity. We performed a segmental duplication analysis to explore gene duplication events within the *ChTRX* and *ChTR* gene families (Figure 5a). *ChTRX-f1-2*, *ChFTRB-2*, and *ChFTRB-3* are located on unnamed chromosomes, not on the known 16 chromosomes of *C. hupingshanesis*. Therefore, intra-genomic collinearity analysis was performed on 73 *ChTRX* family members and 10 *ChTR* family members. We identified 60 collinear gene pairs across different chromosomes, indicating segmental duplication (Figure 5a). Chromosomes 8 and 9 had the highest number of duplicated *ChTRX* gene pairs, with 16 pairs each, followed by chromosomes 14 and 7, with 13 and 11 pairs, respectively. Chromosome 4 contained 8 pairs; chromosomes 2, 3, 10, and 11 each had 7 pairs; chromosomes 1, 5, 6, 12, and 13 had 5 pairs; and chromosome 16 had 3 pairs (Table S4). These findings suggest segmental duplication significantly expanded the *ChTRX* and *ChTR* gene families. Inter-species collinearity analysis involving the dicot *A. thaliana*, the monocot wheat, and *C. hupingshanensis* (Figure 5b,c) showed that *C. hupingshanensis* is more closely related evolutionarily to *A. thaliana* than to *T. aestivum*. Additionally, *C. hupingshanensis* has more chromosomes than *A. thaliana*, indicating that substantial chromosomal rearrangements, such as breaks and fusions, occurred during its evolution.

### 3.5. Analysis of Cis-Acting Elements in ChTRX and ChTR Gene Families

**Figure 6:**
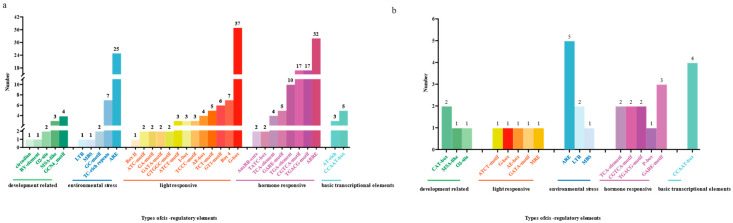
Analysis of *cis*-acting elements in *ChTRX* and *ChTR* gene families. (**a**,**b**) Display quantitative statistics of major cis-acting elements, with key abbreviations defined as follows: ARE (Anaerobic Response Element), G-box (Light-Responsive Element), Box 4 (Component of Conserved Light-Responsive DNA Module), AE-box (Light-Responsive Module Component), ABRE (Abscisic Acid Response Element), and TGACG-motif/CGTCA-motif (MeJA-Responsive Elements).

Gene transcription is controlled by cis-elements located in promoter regions upstream of coding sequences [47]. To predict cis-acting elements, the 2 kb upstream promoter regions of 74 *ChTRXs* and 12 *ChTRs* were analyzed using the online database PlantCARE. Data analysis revealed 33 cis-elements or binding sites in *ChTRX* promoters and 17 in *ChTR* promoters. These elements fall into four main categories: light-responsive, hormone-responsive, growth and development-related, and environmental stress-related, along with core transcription elements (Figure 6a and Figure S1a). The most common were light-responsive, phytohormone-regulatory, and stress-related ecological elements (Figure 6b and Figure S1b). Notably, the Anaerobic Response Element (ARE), associated with environmental stress, was widespread across both gene families. Conversely, the drought-inducible MYB binding site and low-temperature-responsive elements were found in only a few family members. Typical MeJA-responsive motifs, such as TGACG and CGTCA, were also abundant in both families, linked to the jasmonic acid pathway and possibly regulating defence genes in response to biotic and abiotic stresses. The promoter regions of both *ChTRXs* and *ChTRs* were rich in light-responsive elements, including motifs such as the G-box, Box 4, and AE-box. The higher number of these in *ChTRXs* suggests a core regulatory role in photosynthesis. This dense, light-responsive element indicates an efficient light adaptation mechanism in *C. hupingshanensis*, a shade-tolerant plant, that enables the adjustment of photosynthetic enzymes under low light conditions. The Abscisic Acid Response Element (ABRE), essential in ABA signalling, was frequently found in *ChTRX* genes, underscoring its role in drought and salinity stress responses. These cis-acting elements work together to regulate gene expression in response to various stresses, promoting plant survival and adaptability (Figure 6).

### 3.6. Expression Analysis of ChTRXs and ChTRs in Leaves Under Se Stress

**Figure 7:**
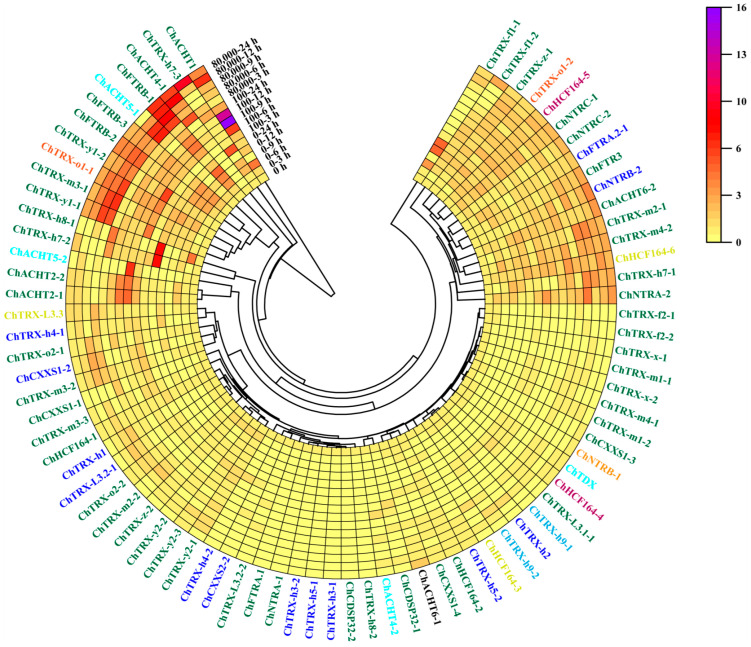
Heatmap showing the expression levels of *ChTRXs* and *ChTRs* in leaves under different Se treatments (0, 100, and 80,000 μg Se·L^−1^). Different colours represent different subcellular organelles: green: Chloroplast, orange: mitochondrion, blue: Cytoplasm, magenta: Endoplasmic Reticulum, cyan: Nucleus, grass green: Plasma membrane, black: Extracellular, sky blue: Cytoskeleton. Gene expression was normalized using *ChActin* as the reference gene, with the expression level at 0 h set to 1.

To investigate the response of *ChTRX* and *ChTR* genes to Se stress, the expression levels of these genes in *C. hupingshanensis* seedlings were analyzed using quantitative real-time PCR (qRT-PCR) technology. This experiment examined their expression levels in leaves under both low and high concentrations of selenium stress (Figure 7) and performed significance analysis (Table S6). Cluster analysis of gene expression under different Se concentrations revealed that genes at the ends of the cluster responded strongly. In contrast, those in the middle section showed little to no expression. Among the numerous responsive *ChTRX* and *ChTR* genes, those located in the chloroplast responded more strongly than genes in other subcellular organelles, indicating the central role of the chloroplast in regulating redox balance under stress. In the typical thioredoxins, the chloroplast-localized *ChTRX-y1-1* and *ChTRX-y1-2*, *ChTRX-m3-1*, and the mitochondria-localized *ChTRX-o1-1* exhibited extremely high responses at both 100 and 80,000 μg Se·L^−1^ treatments. *ChTRX-y1-1* and *ChTRX-y1-2* reached their peak response values, 6.26-fold and 4.66-fold increases after 9 h of treatment with 80,000 μg Se·L^−1^. *ChTRX-m3-1* and *ChTRX-o1-1* were upregulated 6.84-fold and 5.84-fold, respectively, after 6 h of treatment with 80,000 μg Se·L^−1^. Among the atypical thioredoxins, the chloroplast-localized *ChACHT1* was significantly upregulated 15.87-fold after 9 h of treatment with 100 μg Se·L^−1^. *ChACHT4-1*, *ChACHT5-1*, and *ChHCF164-4* also showed varying degrees of response under different Se treatment conditions. The gene expression analysis results demonstrate that *ChTRX* genes are dynamically regulated by Se stress.

Under blank control conditions (0 μg Se·L^−1^), analysis of *ChTR* genes expression revealed that the transcript levels of *ChNTRB-2*, *ChNTRC-1*, and *ChNTRC-2* in the leaves of *C. hupingshanensis* seedlings exhibited dynamic fluctuations over the 24 h (Figure 7). When seedlings were treated with 100 and 80,000 μg Se·L^−1^, the expression responses of *ChFTRB-1*, *ChFTRB-2*, and *ChFTRB-3* genes were stronger, with the change in *ChFTRB-1* expression being particularly significant. This indicates that *ChTR* genes can also be expressed under high Se stress.

### 3.7. Expression Analysis of ChTRXs and ChTRs in Roots Under Se Stress

**Figure 8:**
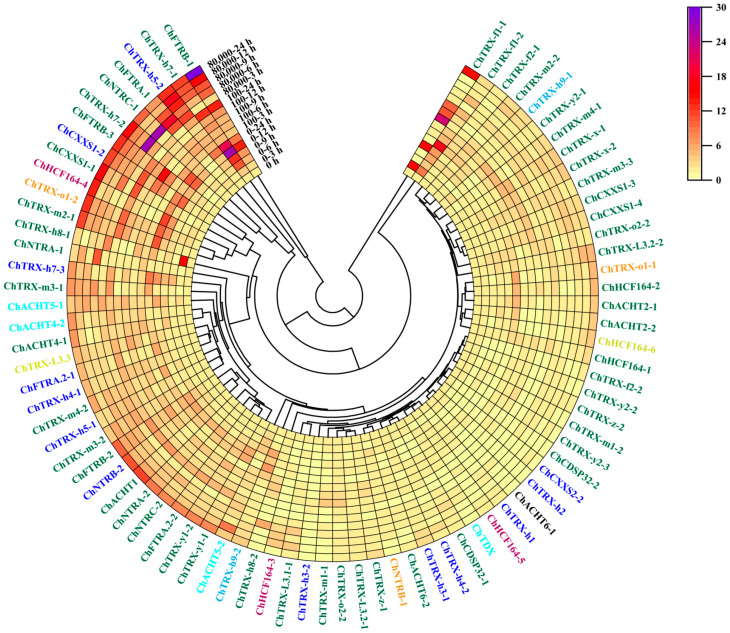
Heatmap showing the expression levels of *ChTRXs* and *ChTRs* in the root under different Se treatments (0, 100, and 80,000 μg Se·L^−1^). Different colours represent different subcellular organelles: green: Chloroplast, orange: mitochondrion, blue: Cytoplasm, magenta: Endoplasmic Reticulum, cyan: Nucleus, grass green: Plasma membrane, black: Extracellular, sky blue: Cytoskeleton. Gene expression was normalized using *ChActin* as the reference gene, with the expression level at 0 h set to 1.

The analytical method for root gene expression was consistent with that used for leaves (Figure 8, Table S7). Cluster analysis of root gene expression revealed that genes at the cluster’s ends responded strongly to Se stress. Under stress conditions, the overall responsiveness of *ChTRX*/*ChNTR* genes in roots was significantly higher than in leaves. In terms of expression levels, under 80,000 μg Se·L^−1^ stress, gene expression in roots was up-regulated by up to 25.96-fold, while in leaves, it reached a maximum up-regulation of 8.27-fold. Regarding the proportion of responsive genes, 31.25% of genes responded in leaves, compared to 52.5% in roots under Se stress. At 80,000 μg Se·L^−1^ treatment for 24 h, most genes exhibited a strong positive response, indicating that root *ChTRXs* and *ChTRs* genes play a significant role under Se stress. At the 100 μg Se·L^−1^ treatment, *ChTRX-f1-1* was significantly upregulated (22.53-fold) at 9 h. In contrast, *ChTRX-m1-1*, *ChTRX-m3-1*, *ChTRX-m3-2*, *ChTRX-m4-2*, and *ChTRX-h7-2* exhibited sustained upregulation throughout the 24 h treatment period. Among these, *ChTRX-h7-2* exhibited the strongest response, reaching a 25.74-fold increase at 6 h. Under 80,000 μg Se·L^−1^ stress, the expression patterns of genes displayed complex dynamic changes. Chloroplast-localized *ChACHT4-1*, *ChCXXS1-1*, *ChTRX-L3.2-2* (*ChTRX-Like3.2-2*), endoplasmic reticulum-localized *ChHCF164-4*, and nucleus-localized *ChACHT4-2* showed a pattern of strong → weak → strong response over time.

*ChTR* genes in the roots also exhibited distinct responses to Se stress. Low Se stress activated 10 *ChTR* genes, including the newly responsive *ChNTRA-1* and *ChFTRA.1*. *ChFTRB-1* and *ChFTRB-2* were strongly upregulated at 24 h, with 13.42- and 6.71-fold increases, respectively. In contrast, *ChFTRB-3* peaked at 9 h with a 15.06-fold upregulation. High stress enhanced the responses, particularly among chloroplast-localized genes. *ChNTRC-1* and *ChFTRB-1* showed maximum induction at 6 h, upregulation of 25.96-fold and 29.51-fold, respectively. *ChFTRA.1* was consistently upregulated more than 10-fold between 3 and 9 h. Comparative analysis revealed that all genes, except *ChNTRB-1*, were differentially expressed. *ChFTRA.1* and *ChFTRB-3* were specifically upregulated under Se stress, indicating selenite-mediated regulation of *ChTRs* activity.

### 3.8. Secondary and Tertiary Structure Prediction of ChTRXs and ChTRs Proteins

Combining the alignment of ChTRX and ChNTR protein sequences (Figure S2) with gene expression analysis results in leaves and roots under Se stress; 5 ChTRXs and 4 ChNTRs were selected to predict their secondary and tertiary structures. Secondary structure analysis was performed using the SOPMA online server for the 5 ChTRXs (ChTRX-y1-1, ChTRX-y1-2, ChTRX-m3-1, ChACHT1, and ChACHT4-1) and the 4 ChNTRs (ChNTRA-1, ChNTRB-1, ChNTRC-1, and ChNTRC-2). The results indicated that the protein sequences of both gene families are primarily composed of alpha helices, beta sheets, extended strands, and random coils. Among these, alpha-helices and random coils were predominant, while beta-sheets were less frequent. This suggests structural differences among these proteins, as detailed in Table 1. The protein sequences of ChTRXs and ChNTRs were submitted to the AlphaFold3 online server to visualise the tertiary structures better. AlphaFold3 predicts the 3D protein structure based on the amino acid sequence (Figure S3). A predicted TM-score (pTM) > 0.5 indicates that the overall predicted protein structure is likely similar to the actual structure. The pTM scores for all proteins except ChNTRC-1 exceeded 0.5, indicating that the structures predicted by AlphaFold3 are reliable and suitable for further investigation.

### 3.9. Simulation of the Thioredoxin System-Mediated Redox Regulation of the Se Metabolic Pathway ChAPK/ChAPR in C. hupingshanensis

**Figure 9:**
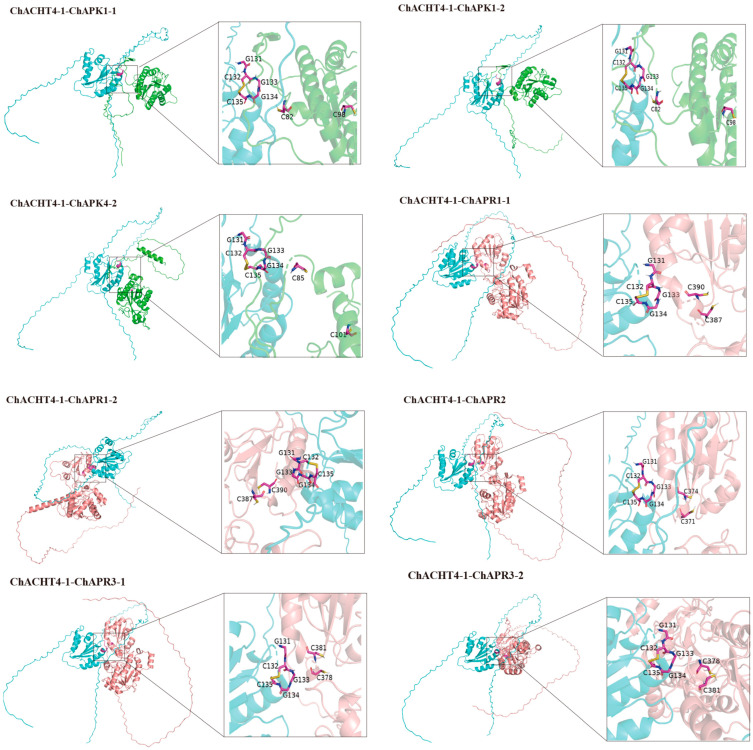
Docking of reduced ChTRX with oxidised ChAPK/ChAPR proteins. The **left** panel displays the overall view, while the **right** panel shows a detailed view of the redox-active site. Proteins are displayed in surface representation; amino acid residues in the redox active site are shown in purple; disulfide bonds in yellow.

Based on existing research, members ChAPK1-1, ChAPK1-2, and ChAPK4-2 of the APK family possess sites for disulfide bond formation, while all 5 members of the APR family contain sites for disulfide bond formation [48]. Since TRX regulates target proteins’ redox state through disulfide bonds in its redox-active center, this study used the HADDOCK [49] website to predict the redox interactions between TRX and APK/APR. Expression analysis under selenium stress identified five TRX genes with high responsiveness to abiotic stress: ChTRX-y1-1, ChTRX-y1-2, ChTRX-m3-1, ChACHT1, and ChACHT4-1. These were selected for protein-protein docking with members of the APK and APR families. Protein modeling was performed using AlphaFold3, and the oxidized states of the target proteins were generated using Maestro. Docking simulations with reduced ChACHT4-1 revealed that the disulfide bonds at the active sites of all eight target proteins—including ChAPK1-1, ChAPK1-2, ChAPK4-2, ChAPR1-1, ChAPR1-2, ChAPR2, ChAPR3-1, and ChAPR3-2—were reduced. Concurrently, a new disulfide bond formed between Cys^132^ and Cys^135^ within the GCGGC active site of ChACHT4-1 (Figure 9 and Figure S4). These results indicate that ChTRX can regulate the reduction process of APK and APR proteins.

**Figure 10:**
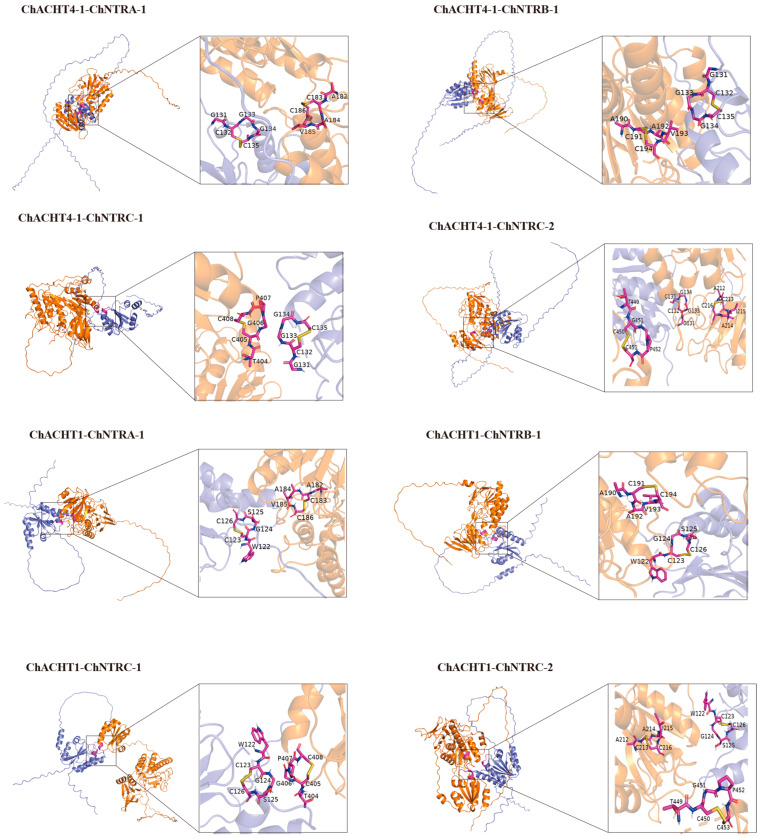
Docking of oxidised ChTRXs with reduced ChNTRs. The **left** panel displays the overall view, while the **right** panel shows a detailed view of the redox-active site. Proteins are displayed in surface representation; amino acid residues in the redox-active site are shown in purple; disulfide bonds are shown in yellow.

**Figure 11:**
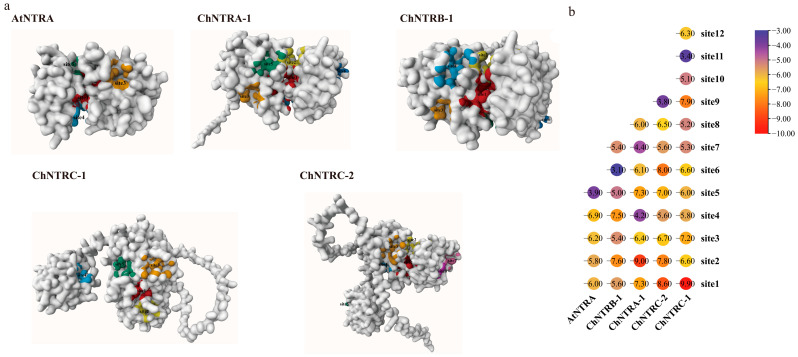
Auto Dock Vina performed molecular docking between ChNTR proteins and FADH2. (**a**) Illustrates the predicted ligand-binding sites on these proteins, visualised using PrankWeb website. (**b**) Displays the binding energies of AtNTRA, ChNTR with FADH2.In the heatmap, the horizontal axis (bottom) lists the gene names, the vertical axis represents the ligand-binding sites, and the values indicate the binding energies (in kcal·mol^−1^) derived from protein-ligand docking.

In subsequent simulations, oxidized ChACHT4-1 and ChACHT1 were docked with reduced ChNTR. The results showed disulfide bonds formed between the cysteine residues at the active site of ChNTR (Figure 10 and Figure S5). Similar regulatory patterns were observed for ChTRX-y1-1, ChTRX-y1-2, and ChTRX-m3-1 (Figure S6), suggesting that ChNTR may reduce oxidized ChTRX. We initially performed homology modeling predictions for the ChNTRs using the SWISS-Model website. The results showed that the template with the highest match for ChNTRA-1 and ChNTRB-1 was the NADPH-dependent thioredoxin reductase from *A. thaliana* (PDB ID: 1VDC.1), while the best-matched template for ChNTRC-1 was the NTRC reductase from *Chlamydomonas reinhardtii* (PDB ID: 7P9D.1). Both of these protein crystal structures contain the cofactor FAD. This cofactor mediates electron transfer to the disulfide bond in the active site of thioredoxin reductase, providing electrons for the subsequent reduction process of TRX [50,51]. This characteristic offers crucial insights for studying the functional mechanism of the ChNTRs. Subsequently, potential binding site predictions conducted using the Prankweb server revealed that AtNTRA (PDB ID: 1VDC.1) and the 4 ChNTR proteins each possess 5 to 12 candidate binding pockets for FADH2/FAD (Figure 11a). Potential binding site predictions performed via the Prankweb server revealed that both AtNTRA and 4 ChNTR proteins possess 5–12 candidate binding pockets for FADH2/FAD. Molecular docking using AutoDock Vina showed negative binding energies between all predicted sites and FADH2 (Figure 11b), indicating likely interactions. The optimal binding affinities varied among ChNTR isoforms: ChNTRC-1 and ChNTRC-2 exhibited the strongest binding at site 1 (−8.6 and −9.9 kcal·mol^−1^), while ChNTRA-1 and ChNTRB-1 showed the highest affinity at site 2 (−7.6 and −9.0 kcal·mol^−1^). By comparison, AtNTRA had a maximum binding energy of −6.9 kcal·mol^−1^ at site 4, suggesting generally stronger binding between ChNTR proteins and FADH2. The predicted redox center of AtNTRA at site 1 showed a binding energy of −6.0 kcal·mol^−1^. This meets the criterion that “a binding energy lower than −5 kcal·mol^−1^ indicates strong binding and a high likelihood of reaction occurrence.” The result is highly consistent with previously reported experimental conclusions [50], validating the feasibility of the predictive method and docking results in this study. Furthermore, ChNTRA-1, ChNTRB-1, and ChNTRC-2 all contain the conserved “ACVAC” redox motif at site 1, with binding energies below −5 kcal·mol^−1^, supporting a conserved catalytic mechanism with AtNTRA and the hypothesis that FADH2 may function by binding to this redox-active site in ChNTR proteinsChNTRB-1 showed the highest affinity at Site 2, with binding energies of −7.6 kcal·mol^−1^ and −9.0 kcal·mol^−1^. Furthermore, the redox-active motif “ACVAC” in ChNTRA-1, ChNTRB-1, and ChNTRC-2 was located within Site 1, where all binding energies were below −5 kcal·mol^−1^. These findings suggest that FADH2, as a cofactor of ChNTR, may reduce the redox-active site of ChNTR, providing support for a potential electron transfer mechanism.

## 4. Discussion

The thioredoxin system is a core regulatory network for maintaining redox homeostasis in plants [52], yet its role in Se hyperaccumulator plants remains insufficiently explored. This study presents the first genome-wide systematic identification and functional analysis of the thioredoxin system in *C. hupingshanensis*, a Se hyperaccumulator. Results reveal that the *C. hupingshanensis* genome encodes 74 *ChTRX* genes and 12 *ChTR* genes (Tables S1 and S2), which exhibit both conserved features [7] and species-specific adaptive characteristics. These traits may be key to the selenium tolerance and hyperaccumulation capability of *C. hupingshanensis*. Phylogenetic analysis classified the *ChTRX* genes into typical and atypical subtypes (Figure 2a), with active site motifs consistent with those reported in *A. thaliana* and other plants [53]. Notably, compared with *A. thaliana* (41 members) [45] and *Vitis vinifera* (40 members) [54], the *ChTRX* family in *C. hupingshanensis* is significantly expanded (74 members), suggesting that gene duplication events may have contributed to genomic adaptation to selenium-rich environments [55]. In particular, root-specific and chloroplast-targeted subtypes showed notable expansion, potentially enhancing redox regulation under selenium stress.

In the promoter regions of *ChTRX* and *ChTR* genes (Figure 6), widespread presence of ARE, ABRE, and MeJA responsive elements indicates their potential role in mediating transcriptional responses to Se-induced oxidative stress. Subcellular localization predictions indicated that nearly 60% of ChTRX proteins are localized in chloroplasts, consistent with the crucial role of the plastid thioredoxin system in mitigating photo-oxidative damage [14,56]. This feature is particularly significant for *C. hupingshanensis*: under high selenium conditions, plants produce more reactive oxygen species, and chloroplast-localized ChTRX proteins may contribute to both ROS scavenging and the maintenance of photosynthetic efficiency. Meanwhile, the spatiotemporally specific responses of ChFTRB-1 and ChNTRC-1 are consistent with previously reported root-specific upregulation of the *ChATPS* gene under Se stress and its involvement in sulfur metabolism regulation [57]. These findings jointly support the hypothesis that the thioredoxin system participates in Se uptake and assimilation processes by coordinating with key enzymes in Se metabolism. This suggests its potential role in integrating Se metabolism and redox regulation to form a unique stress resistance network in Se hyperaccumulators.

Molecular docking simulations revealed that reduced ChACHT4-1 protein can interact with disulfide bonds in the key selenometabolic enzymes ChAPK and ChAPR, suggesting a direct regulatory role in Se assimilation. This finding is consistent with recent studies in *C. hupingshanensis* confirming the importance of APK and APR in Se metabolism and their regulation by redox status [48]. Additionally, the ChNTR protein exhibited strong binding affinity with FADH2, indicating a functional electron transfer mechanism similar to that of the plant thioredoxin system [14]. Concurrently, the recently identified “thioredoxin-mediated desulfuration” process in plants [58] opens new perspectives for understanding how the thioredoxin system integrates diverse redox modifications to finely tune stress responses. However, this study has several limitations: molecular docking was based on predicted protein structures without validation through in vitro enzymatic assays; protein–protein interactions were not confirmed by co-immunoprecipitation (co-IP) or pull-down assays; the functional role of catalytic cysteine residues was not verified via mutagenesis; and quantitative analysis of Se species transformation was not performed. These limitations preclude the establishment of definitive causal relationships among the factors involved.

In conclusion, this study provides a preliminary reference for understanding the function of the thioredoxin system in Se hyperaccumulating plants. Although using a high concentration of selenite (80,000 μg Se·L^−1^) in the treatment effectively revealed gene expression responses at this level, the substantial variation in natural Se concentrations may affect the generalizability of the conclusions. In subsequent research, we plan to further investigate plant responses under different concentration gradients and analyse specific Se species, aiming to provide a more comprehensive interpretation of the regulatory mechanisms of Se on this system and offer richer perspectives for related studies.

## 5. Conclusions

This study presents the first systematic investigation of the *TRX* and *TR* gene families in *C*. *hupingshanensis*. Through genome-wide identification, 74 *ChTRX* genes and 12 *ChTR* genes were obtained, and subsequent bioinformatic analyses characterised the fundamental features of these gene families, providing initial predictions of the thioredoxin-mediated Se stress response mechanisms in hyperaccumulator plants, advancing research in the field. Furthermore, we observed that chloroplast-localized ChTRX and ChTR members showed significant responses to Se stress, implying a central role of the chloroplast in Se tolerance in this species. This finding highlights the importance of organelle-specific regulatory hubs. Molecular docking simulations further predicted that the thioredoxin system can dynamically modulate the redox status of key Se metabolic enzymes (ChAPKs/ChAPRs), suggesting that in hyperaccumulators, the thioredoxin system may not only serve general antioxidant functions but could also specifically regulate Se metabolic pathways. Although this study offers preliminary clues regarding the involvement of ChTRX in plant Se tolerance and the link between the thioredoxin system and Se metabolism, certain limitations remain—such as the unconfirmed causal role of ChTRX in Se tolerance and the lack of in vitro validation of the regulatory mechanisms between the thioredoxin system and Se metabolism. Overall, the structural bioinformatics approaches employed here enhance the understanding of redox regulatory networks in specialised plants and provide new ideas for enhancing plant tolerance to abiotic stresses.

## Figures and Tables

**Table 1:** Analysis of Model Parameters and Secondary Structure for ChTRX and ChNTR Proteins.

Gene Name	pTM	Alpha Helix	Beta Turn	Extended Strand	Random Coil
*ChTRX-y1-1*	0.56	27.91%	4.65%	11.63%	55.81%
*ChTRX-y1-2*	0.60	29.81%	4.97%	12.42%	52.80%
*ChTRX-m3-1*	0.60	27.59%	4.02%	16.67%	51.72%
*ChACHT1*	0.57	30.13%	3.49%	9.17%	57.21%
*ChACHT4-1*	0.54	26.26%	3.24%	7.91%	62.59%
*ChNTRA-1*	0.83	29.30%	7.32%	21.41%	41.97%
*ChNTRB-1*	0.83	30.38%	6.45%	20.97%	42.20%
*ChNTRC-1*	0.46	38.54%	5.62%	17.50%	38.33%
*ChNTRC-2*	0.66	33.33%	6.48%	18.86%	41.33%

## Data Availability

The datasets generated and analyzed during the current study are available from the corresponding authors upon reasonable request.

## Supplementary Materials

The following supporting information can be downloaded at: https://www.mdpi.com/article/10.3390/biology14101404/s1. Figure S1: The number and types of cis-regulatory elements identified in *ChTRX* and *ChTR* gene families; Figure S2: Multiple sequence alignment of full-length TRX and TR proteins in *C.hupingshanensis*; Figure S3: Protein structural models of ChTRXs and ChNTRs; Figure S4: Protein models of reduced ChACHT4-1 and oxidized ChAPKs/ChAPRs; Figure S5: Protein models of oxidized ChTRXs and reduced ChNTRs; Figure S6: Docking of oxidised ChTRXs with reduced ChNTRs; Table S1: Identification of basic physicochemical properties of *ChTRXs* gene family members; Table S2: Identification of basic physicochemical properties of *ChTRs* gene family members; Table S3: Coding sequences and protein sequences of *ChTRXs* and *ChTRs* genes; Table S4: Details of *ChTRXs* and *ChTRs* gene pairs involved in gene duplication events; Table S5: Primers for qRT-PCR analysis of *ChTRX* and *ChTR* genes; Table S6: Statistical Analysis of Temporal Differential Expression of *ChTRX* and *ChTR* Genes in Leaves Under Selenium Stress; Table S7: Statistical Analysis of Temporal Differential Expression of *ChTRX* and *ChTR* Genes in Roots Under Selenium Stress.
