# Supplementary material for: Genome-Wide Thioredoxin System in Cardamine hupingshanensis: Role in Se Stress and Metabolism
Source: Biology (Basel). 2025 Oct 13;14(10):1404. doi: 10.3390/biology14101404 (PMC12561512; doi:10.3390/biology14101404)
Supplement: Supplementary file 1 [file biology-14-01404-s001.zip › Supplementary Figure S1-S6.pdf]

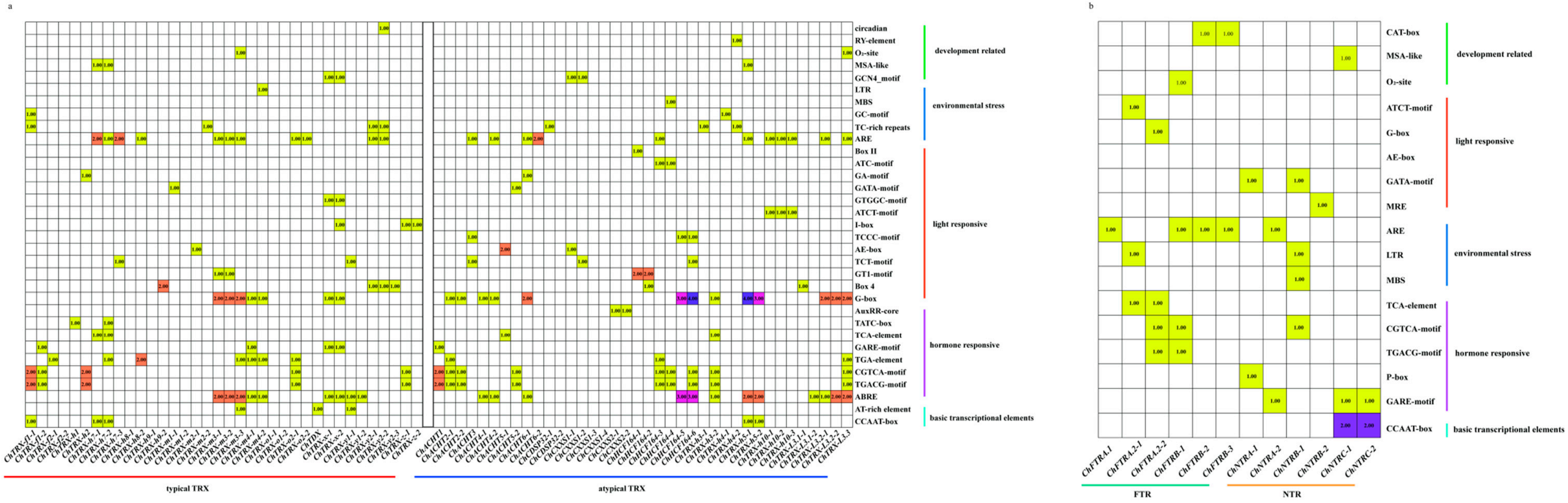


**Figure S1.** The number and types of cis-regulatory elements identified in *ChTRX* and *ChTR* gene families.

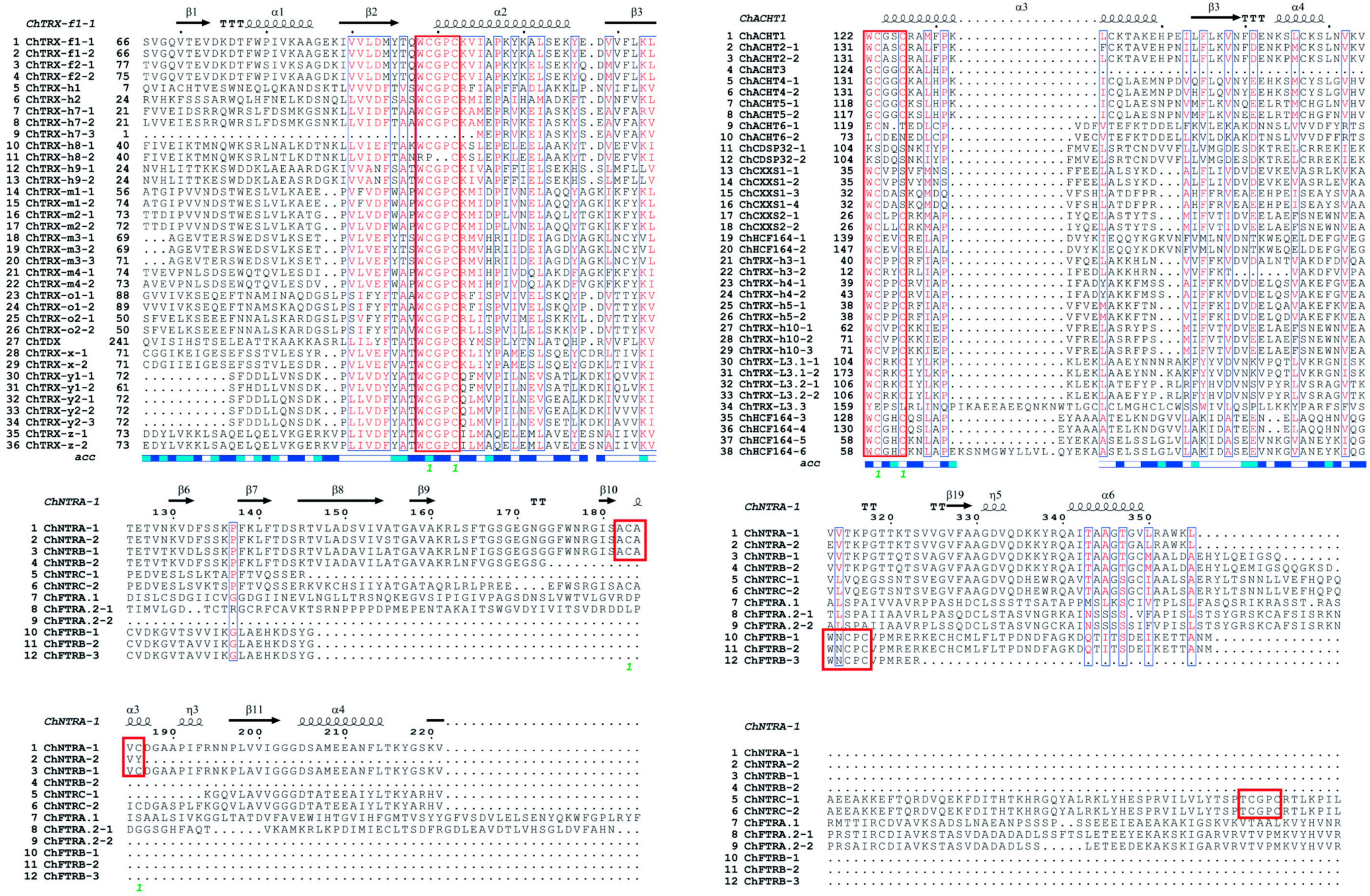

**Figure S2.** Multiple sequence alignment of full-length TRX and TR proteins in *C.hupingshanensis.* The red boxes indicate the redox active sites of ChTRXs and ChTRs.

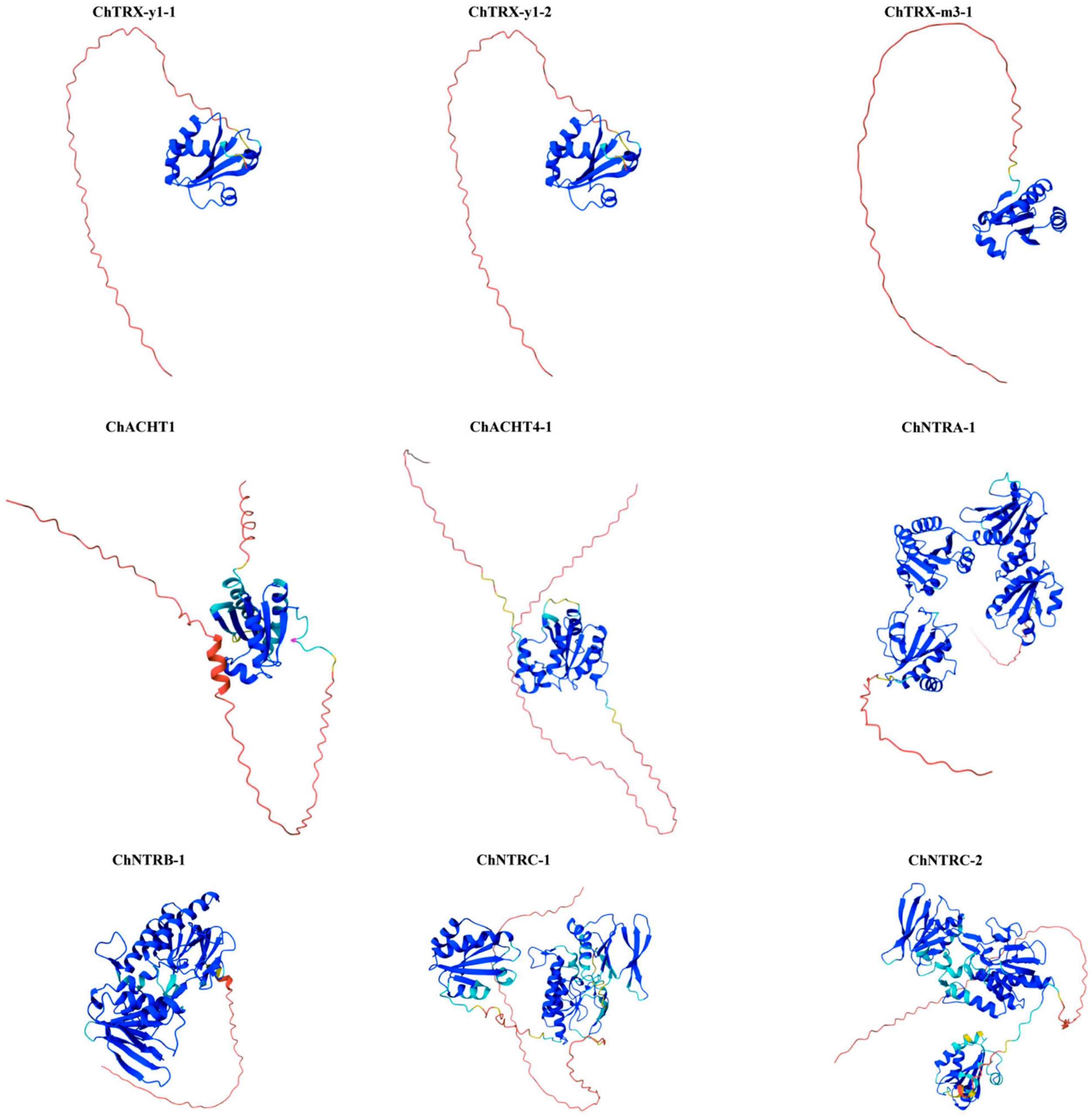


**Figure S3.** Protein structural models of ChTRXs and ChNTRs.

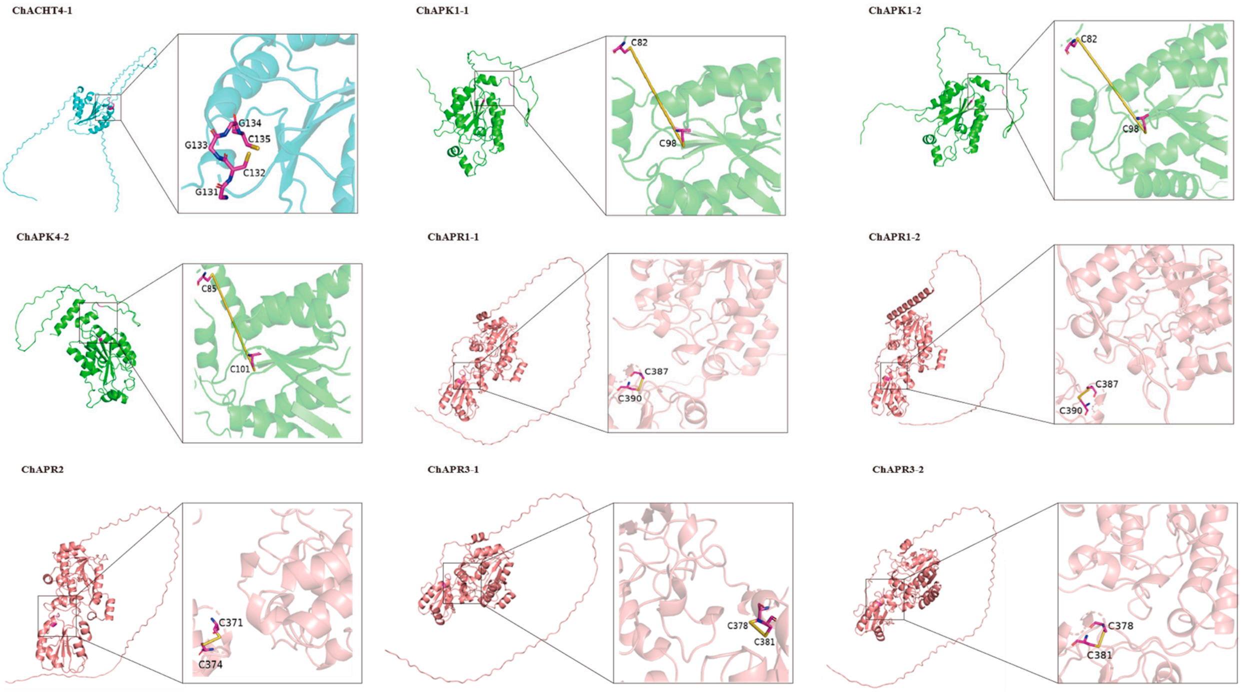


**Figure S4.** Protein models of reduced ChACHT4-1 and oxidized ChAPKs/ ChAPRs.

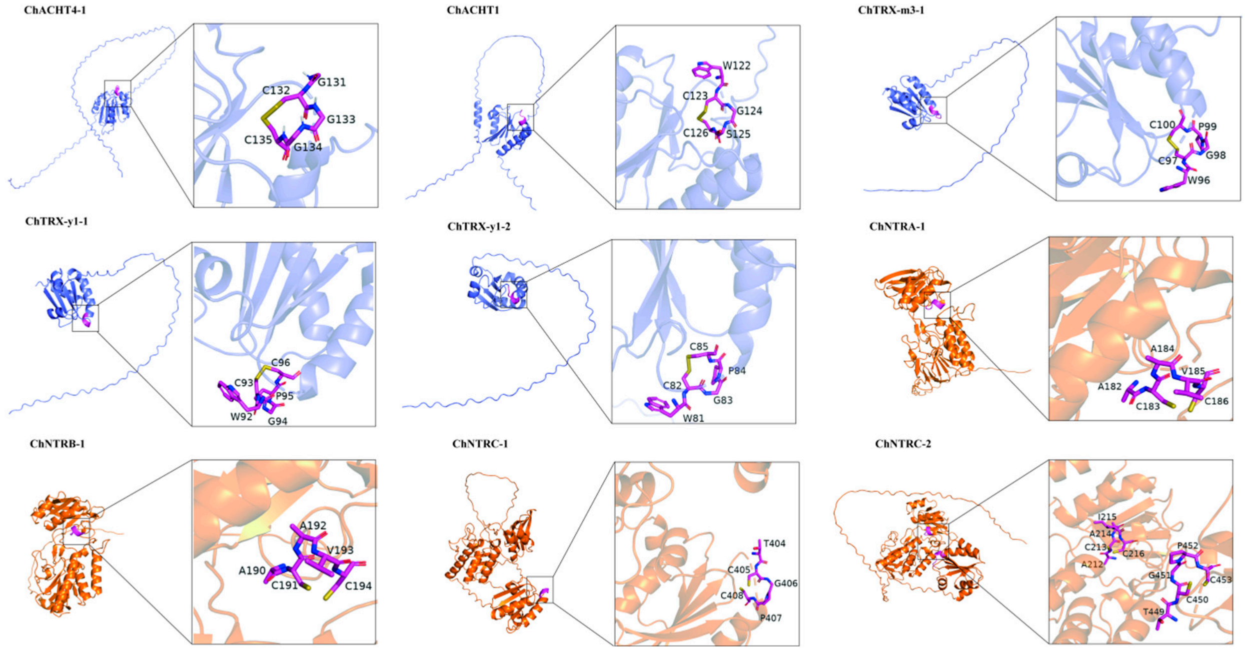


**Figure S5.** Protein models of oxidized ChTRXs and reduced ChNTRs.

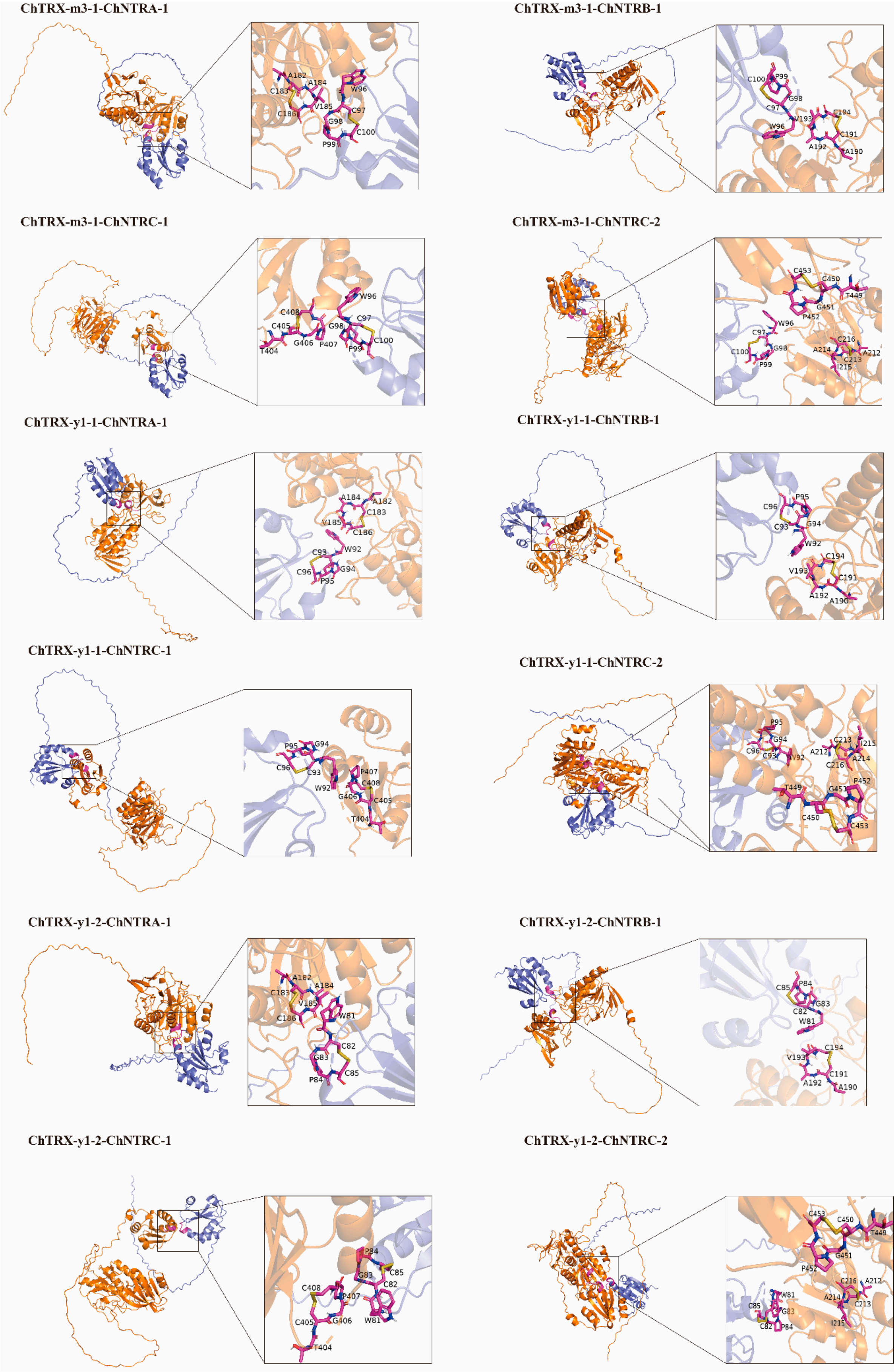


**Figure S6.** Docking of oxidised ChTRXs with reduced ChNTRs.
